# Combination of peroxisome proliferator-activated receptor α/γ agonists may benefit type 2 diabetes patients with coronary artery disease through inhibition of inflammatory cytokine secretion

**DOI:** 10.3892/etm.2013.891

**Published:** 2013-01-10

**Authors:** JINRU WEI, QUAN TANG, LIJUAN LIU, JIANBIN BIN

**Affiliations:** Department of Cardiology, The Fifth Affiliated Hospital of Guangxi Medical University, Liuzhou, Guangxi 545006, P.R. China

**Keywords:** diabetes mellitus, coronary heart disease, peroxisome proliferator-activated receptor α, peroxisome proliferator-activated receptor γ, inflammatory cytokine

## Abstract

Patients with type 2 diabetes mellitus (T2DM) have a higher risk of cardiovascular disease (CVD). Peroxisome proliferator-activated receptors (PPARs) play an important role in the regulation of energy homeostasis. Therefore, we aimed to observe the effects of combined PPARα/γ agonists on T2DM patients with coronary artery disease (CAD). Patients were randomly divided into a rosiglitazone (RSG) group (n=20), a bezafibrate (BEZ) group (n=20), a combination of RSG and BEZ group (n=20) and a control group (n=20). Plasma C-reactive protein (CRP) and monocyte chemoattractant protein-1 (MCP-1) were measured by enzyme-linked immunosorbent assay before and 12 weeks after treatment. Fasting blood glucose (FBG), fasting insulin, insulin resistance index (IRI), hemoglobin A1c (HbA1c), lipid levels and body mass index were also investigated. At the end of the treatment, FBG, insulin, IRI, HbA1c and triglyceride levels decreased and the level of high-density lipoprotein cholesterol increased in the RSG, BEZ and combination groups. A decrease in low-density lipoprotein cholesterol was only observed in the combination group. Although the total cholesterol levels in all groups decreased, no significant difference was noted. The levels of CRP and MCP-1 were reduced in patients in the RSG, BEZ and combination groups. In addition, RSG, BEZ and the combination of RSG and BEZ also inhibited MCP-1 secretion. The combination of RSG and BEZ was more efficient than RSG or BEZ alone in downregulating cytokines. In conclusion, our results suggest that a combination of RSG and BEZ may be more efficient than RSG or BEZ alone in the treatment of T2DM patients with CAD.

## Introduction

Patients with type 2 diabetes mellitus (T2DM) have a higher risk of cardiovascular disease (CVD), including coronary artery disease (CAD), the main cause of premature morbidity and mortality in these patients (1,2). Inflammation has been indicated to be an important contributor to the development of T2DM and CAD, not only by promoting atherogenesis, but also by inducing insulin resistance (IR) and β-cell impairment.

Peroxisome proliferator-activated receptors (PPARs) belong to the nuclear receptor superfamily and are ligand-activated transcription factors. There are three isoforms, α, β and γ (3). PPARs play an significant role in the regulation of energy homeostasis by regulating the expression of a variety of genes involved in lipid and carbohydrate metabolism (4). Data from murine models also suggest that PPAR agonists have independent anti-atherosclerotic actions, including the suppression of vascular inflammation, oxidative stress and activation of the renin-angiotensin system (RAS) (5). PPAR agonists reduce the production of tumor necrosis factor (TNF)-α, interleukin (IL)-6 and IL-1β (6,7). PPARα activation indirectly modulates inflammatory components in high-density lipoprotein (HDL), including apolipoprotein A1 (apoA1), serum amyloid A and paraoxonase-1 (8). PPARγ activators inhibit the expression of matrix metalloproteinase (MMP)-9 in human vascular smooth muscle cells and macrophages (9). PPARγ activators also inhibit the production of TNF-α, IL-6 and IL-1β by activated monocytes (10). A number of these effects are mediated by transrepression of the nuclear factor-κB (NF-κB) and activator protein-1 (AP-1) dependent pathways (7,11).

The dual PPARα/γ agonists were developed due to the apparent efficacy of PPARα and PPARγ agonists individually on metabolic control, providing the possibility of optimizing the metabolic and anti-atherosclerotic actions through activation of the two receptors. These dual agonists, including ragaglitazar and muraglitazar, demonstrate a higher affinity for PPARγ than conventional thiazolidinediones and are highly effective at improving metabolic parameters (12,13). However, the effects of these agents on atherogenesis are not clearly understood. Treatment with a dual PPARα/γ agonist, compound 3q, notably increased atherosclerosis in control apoE knockout mice (14), while PPARγ and α agonists used alone in this model were protective (15,16). Furthermore, treatment with a dual PPAR agonist in mice resulted in plaque accumulation accompanied by an increase in aortic gene expression of the pro-inflammatory molecules, P-selectin, CD36, vascular cell adhesion molecule 1 (VCAM-1) and monocyte chemoattractant protein-1 (MCP-1) and increased macrophage infiltration, an effect not observed with the single PPAR agonists, rosiglitazone (RSG) or gemfibrozil (14). One study suggested that dual PPARα/γ agonists are also associated with an increased risk of adverse cardiovascular events when used by individuals with diabetes (17). Therefore, there is a long distance from the bench to the clinic for the dual PPARα/γ agonists and a new strategy that may benefit T2DM patients with CAD should be explored.

RSG, a PPARγ agonist typically used to treat T2DM patients (18,19), significantly reduces homocysteine-induced reactive oxygen species and the secretion of MCP-1 and IL-8 in human monocytes. It also significantly decreases plasma C-reactive protein (CRP) and MCP-1 levels in T2DM patients with CAD (20). Bezafibrate (BEZ), a PPARα agonist and an effective drug in the treatment of dyslipidemia, has been shown to be effective in the primary prevention of cardiovascular events (21). Therefore, we combined these PPARα and PPARγ agonists in the treatment of T2DM patients with CAD, with the expectation of greater efficacy and other advantages.

## Materials and methods

### Subjects

Eighty T2DM patients with CAD were enrolled from the Department of Cardiovascular, The Fifth Affiliated Hospital of Guangxi Medical University, China, from January 2006 to December 2008. T2DM was defined as the level of glucose, including fasting blood glucose (FBG) ≥7.0 mmol/l, casual plasma glucose ≥11.1 mmol/l or plasma glucose ≥11.1 mmol/l, 2 h after an oral glucose tolerance test (OGTT).

T2DM patients with CAD, including angina pectoris and myocardial infarction, were diagnosed according to the criteria set out in the American College of Cardiology/American Heart Association (ACC/AHA) guidelines (22). Coronary angiography confirmed at least one stenosis >50%. The exclusion criteria were: complications with infectious or inflammatory diseases or impaired liver or kidney function; coexisting heart failure [New York Heart Association (NYHA) class III–IV]; presence of a malignant tumor; hematological diseases; or treatment with protamine zinc insulin or isophane insulin.

### Study design

The patients were randomly assigned to an RSG group (4 mg/day, n=20), a BEZ group (400 mg/day, n=20), a combination group (RSG plus BEZ, n=20) or a control group (conventional therapy, n=20). All patients received conventional therapy (including oral hypoglycemic agents or subcutaneous insulin injection and low dose statins) for DM and CAD for 12 weeks. RSG was purchased from GlaxoSmithKline (Tianjing, China) and BEZ was purchased from Tianlishidiyi, Ltd. (Huaian, China). Inflammatory and metabolic factors were tested before treatment and after 12 weeks in fasting venous blood. This study was approved by the ethics committee of the Fifth Affiliated Hospital of Guangxi Medical University. All subjects gave their written informed consent.

### Blood parameter analysis

Before treatment and at 12 weeks after treatment, venous blood samples were collected and centrifuged immediately at 3,000 rpm for 20 min. The supernatant was collected and stored at −70°C. Plasma CRP and MCP-1 were determined by enzyme-linked immunosorbent assay (ELISA; R&D Systems, Minneapolis, MN, USA) according to the manufacturer’s instructions. FBG was measured by the standard oxidase method and lipids were measured by the standard dry chemistry method. Hemoglobin A1c (HbA1c) was measured by high performance liquid chromatography (HPLC). Insulin was determined by chemiluminescence. The estimate of IR by homeostasis model assessment (HOMA-IR) was as follows: IR = [fasting insulin (Fins; IU/ml) − fasting glucose (mmol/l)] /22.5 (23).

### Responsiveness of monocytes to lipopolysaccharide

Twelve weeks after treatment, venous blood samples were obtained from fasting subjects to evaluate the effect of RSG and BEZ on MCP-1 production induced by low-dose lipopolysaccharide (LPS) in isolated monocytes. Whole blood was separated into peripheral blood mononuclear cells (PBMCs) and neutrophils using NycoPrep™ 1.077 (Life Technologies Co., Carlsbad, CA, USA) and then monocytes were isolated by their adherence to the flasks. Adherent cells were then detached and resuspended in RPMI-1640 medium containing 5% autologous serum. Then, monocytes (5×10^5^) were incubated at 37°C with or without LPS (0.01 *μ*g/ml) for 24 h. The supernatant was harvested and stored at −70°C for further MCP-1 examination.

### Statistical analysis

CRP and MCP-1 were expressed as the median and range and were analyzed by the Wilcoxon matched-pairs signed ranks test. The difference between groups was analyzed by the Mann-Whitney test. Age, body mass index, metabolic factors, including cholesterol, triglycerides (TGs) and glucose were expressed as the mean ± standard deviation. A paired Student’s t-test was used to compare values pre- and post-treatment. One-way analysis of variance (ANOVA) was performed for multiple comparisons followed by Dunnett’s test. P<0.05 (two-tailed) was considered to indicate a statistically significant difference.

## Results

### Baseline demographic characteristics

Clinical and metabolic parameters of the patients are presented in Table I. Age, gender, smoking status, hypertension, FBG, Fins, HOMA-IR, HbA1c, lipid levels and MCP-1 did not demonstrate significant differences among the four groups (P>0.05). The CRP levels in the BEZ and combination groups were higher than those in the RSG and control groups; however, the differences were not statistically significant (P>0.05).

### FBG, Fins, HOMA-IR and HbA1c levels

**Figure 1.**
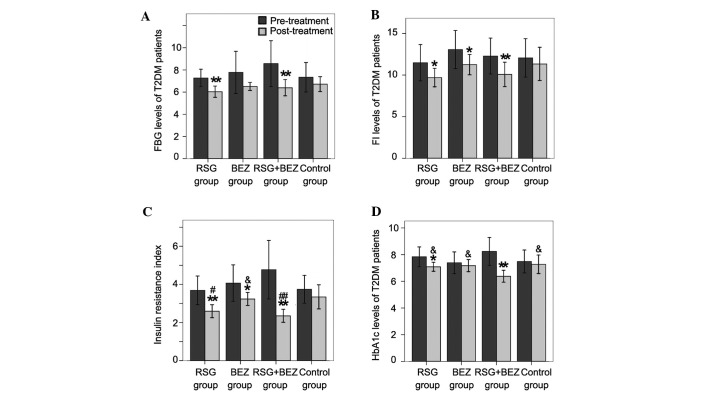
Blood glucose related parameters. (A) FBG, (B) Fins, (C) HOMA-IR and (D) HbA1c levels were determined before and after treatment. Each bar represents the mean ± standard deviation (SD; n=20). ^*^P<0.05, ^**^P<0.01, compared with pre-treatment in the same group; ^&^P<0.05, compared with the combination group; ^#^P<0.05, ^##^P<0.01, compared with the control group. FBG, fasting blood glucose; T2DM, type 2 diabetes mellitus; RSG, rosiglitazone; BEZ, bezafibrate; FI, fasting insulin; HbA1c, hemoglobin A1c; HOMA-IR, insulin resistance by homeostasis model assessment.

The FBG levels in the RSG and combination groups were significantly decreased compared with those before treatment; however, no significant reduction was noted in the BEZ and control groups (Fig. 1A). Fins levels decreased after 12 weeks of treatment with RSG, BEZ or the combination of RSG and BEZ (Fig. 1B). Although a slight decrease was also observed in the control group, no significant difference was detected. Patients in the RSG, BEZ and combination groups demonstrated improved HOMA-IR after 12 weeks of treatment; however, the amelioration was not significant in the control group (Fig. 1C). HOMA-IR in the RSG and combination groups was lower than that in the BEZ and control groups at 12 weeks and no significant difference was observed between the RSG group and the BEZ group. All patient HbA1c was not well-controlled before treatment (Fig. 1D). With RSG and combined treatment for 12 weeks, HbA1c markedly decreased compared with that before treatment; however, the decreases were not significant in the BEZ and control groups. HbA1c in the combination group was almost reduced to a normal level (HbA1c <7%).

### Changes in lipid parameters

**Figure 2.**
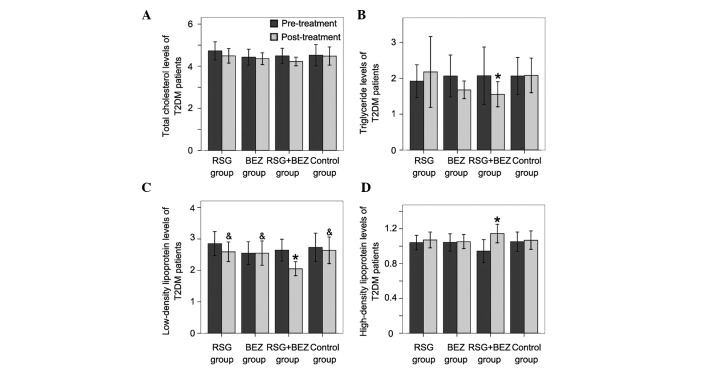
Changes in lipid parameters. (A) TC, (B) TG, (C) LDL and (D) HDL levels were determined before and after treatment. Each bar represents the mean ± standard deviation (SD; n=20). ^*^P<0.05, compared with pre-treatment in the same group; ^&^P<0.05, compared with the combination group. T2DM, type 2 diabetes mellitus; RSG, rosiglitazone; BEZ, bezafibrate; TC, total cholesterol; TG, triglyceride; LDL, low-density lipoprotein; HDL, high-density lipoprotein.

After 12 weeks treatment, total cholesterol (TC) levels in the four groups all decreased; however, no significant differences were observed compared with those before treatment (Fig. 2A). TG levels in the RSG and control groups were slightly increased without significant differences (Fig. 2B), while in the BEZ and combination groups, TG was markedly decreased. The low-density lipoprotein (LDL) level in the combination group was markedly decreased (Fig. 2C); however, the reduction of HDL levels in the other groups was not significant. After 12 weeks of treatment, the HDL level in the combination group was markedly increased, while the HDL levels in the other groups were not significantly elevated (Fig. 2D).

### Plasma CRP and MCP-1 levels

**Figure 3.**
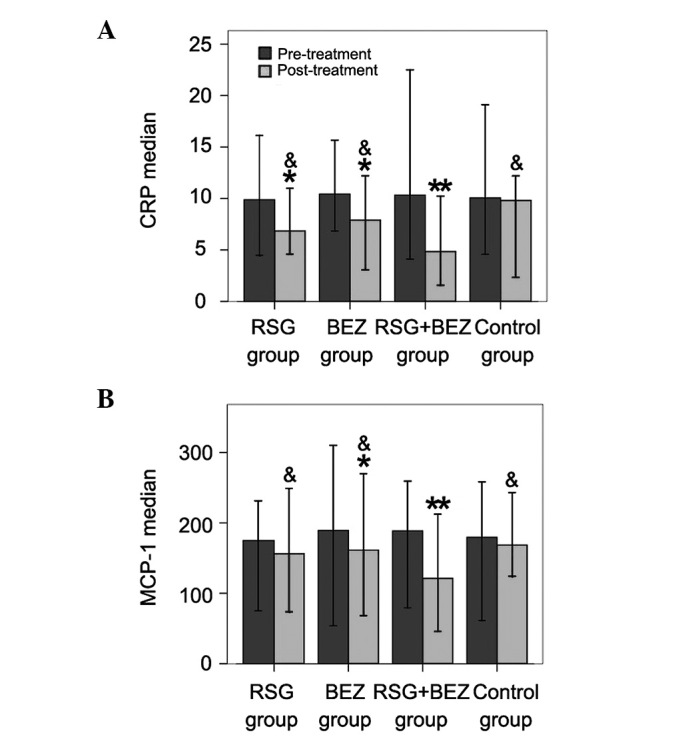
Plasma inflammatory cytokine levels in T2DM patients. (A) CRP and (B) MCP-1 levels were determined before and after treatment. Each bar represents the median and the range (n=20). ^*^P<0.05, ^**^P<0.01, compared with pre-treatment in the same group; ^&^P<0.05, compared with the combination group. CRP, C-reactive protein; RSG, rosiglitazone; BEZ, bezafibrate; MCP-1, monocyte chemoattractant protein-1; T2DM, type 2 diabetes mellitus.

After 12 weeks of treatment, the CRP level in each of the four groups decreased (Fig. 3A). Significant differences in CRP and MCP-1 levels before and after treatment were observed in the RSG, BEZ and combination groups; however, this was not observed in the control group (Fig. 3A and B). Moreover, the levels of CRP and MCP-1 in the combination group decreased more than those in the other treatment groups.

### Chemokine secretion from isolated monocytes in response to LPS

**Figure 4.**
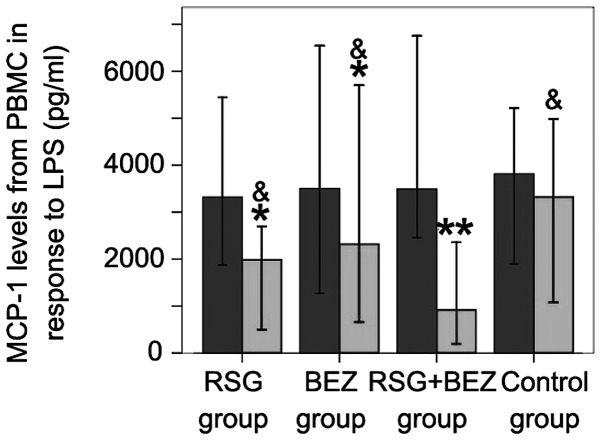
Effect of combined RSG and BEZ on MCP-1 levels secreted by PBMCs in response to LPS. PBMCs in T2DM patients with CAD were incubated with LPS for 24 h. Then, the supernatant was harvested and MCP-1 levels were determined by ELISA. Each bar represents the mean ± standard deviation (SD). ^*^P<0.05, ^**^P<0.01, compared with pre-treatment in the same group; ^&^P<0.05, compared with the combination group. MCP-1, monocyte chemoattractant protein 1; PBMC, peripheral blood mononuclear cell; LPS, lipopolysaccharide; RSG, rosiglitazone; BEZ, bezafibrate; T2DM, type 2 diabetes mellitus; CAD, coronary artery disease; ELISA, enzyme-linked immunosorbent assay.

To test whether monocytes isolated from T2DM patients with CAD demonstrate an enhanced inflammatory response, PBMCs were incubated with LPS (0.01–0.1 *μ*g/ml) for 24 h. LPS at 0.01 lg/ml induced greater MCP-1 secretion by PBMCs from T2DM patients with CAD than from control subjects (Fig. 4). RSG, BEZ and combined RSG and BEZ treatment for 12 weeks significantly decreased low-dose LPS-induced MCP-1 secretion. Combined treatment with RSG and BEZ inhibited the LPS-induced MCP-1 secretion more efficiently than RSG or BEZ used alone.

## Discussion

T2DM is an independent risk factor of CAD. Epidemiological investigation revealed that patients with T2DM had a 2- to 4-fold higher risk than normal individuals of suffering from CAD (24). In the present study, we demonstrated that the combination of PPARγ and PPARα agonists downregulates the blood glucose and lipid levels more efficiently in T2DM patients with CAD than the PPARγ or PPARα agonist alone. The combination of PPARγ and PPARα agonists also inhibited inflammatory cytokine secretion in the T2DM patients with CAD and attenuated the LPS-induced MCP-1 secretion by PBMCs in the T2DM patients. These results suggest that the combination of RSG and BEZ is more effective than RSG or BEZ alone for T2DM patients with CAD, and acts by inhibiting the secretion of inflammatory cytokines from monocytes.

RSG, a well-recognized oral anti-diabetic drug, belongs to the thiazolidinedione class of drugs. RSG increases the expression of glucose transporter-1 and facilitates the uptake of glucose by muscle and fat tissue through its receptors. Thereby, it reduces the output of liver glucose and blood glucose and improves hyperinsulinemia (25,26). RSG also acts as an insulin sensitizer. It inhibits glucagon production and thus increases insulin sensitivity. In the present study, we demonstrated that RSG is capable of alleviating IR and decreasing FBG and HbA1c in T2DM patients. Similar results were observed in the BEZ group. However, when treated with combined RSG and BEZ for 12 weeks, the FBG, HbA1c, insulin and HOMA-IR all reached satisfactory levels. The results indicate that the combination of PPARα/γ agonists improves glucose metabolism more effectively than the PPARγ or PPARα agonist alone.

T2DM patients usually have one or more lipid abnormalities. The main characteristics of dyslipidemia in T2DM patients are an elevated TG level, increased LDL level and decreased HDL level. LDL cholesterol is a major risk factor for CVD in general and in particular in T2DM patients. Statins are extremely effective drugs for reducing the levels of LDL cholesterol. Numerous clinical trials have shown that treatment with statins lowers the rates of heart attack and cardiovascular mortality. Moreover, fibrates reduce TGs and increase the blood levels of HDL cholesterol, which is considered as ‘good’ cholesterol. However, there has been no definitive evidence from previous clinical trials that have used combined fibrate and statin to control blood lipid levels and thereby reduce the risk of heart attack and stroke in T2DM patients. In the present study, we identified that after 12 weeks treatment with combined RSG and BEZ, the TC, TG, LDL-C and HDL-C levels significantly improved. However, no significant changes in the lipid parameters, were observed in the other groups suggesting that the combination of the two drugs produces an improved outcome compared with RSG or BEZ alone.

Chronic inflammation has been shown to be a common precursor of T2DM and CAD, while hyperglycemia and IR may contribute to inflammation. Therefore, inflammation may act as a bridge between T2DM and coronary atherosclerosis (27). The process of PBMC migration into the vascular intima and transformation to foam cells, constitutes the early event of atherosclerosis and is regulated by plasma inflammatory cytokines, including MCP-1 and CRP. MCP-1 is secreted mainly by endothelial cells, vascular smooth muscle cells, monocytes and macrophages. Elevation of the MCP-1 level in CAD patients often indicates the increase and activation of inflammatory cells in the plaque. The increase of MMPs degrades structural matrices in the fibrous cap, which causes plaque rupture and vessel injury. Therefore, MCP-1 plays an important role in the acute coronary syndrome caused by atherosclerosis and plaque rupture (28). PPARs negatively regulate NF-κB and stimulate protein-1 activity, thereby inhibiting inflammatory gene transcription (29). Studies in animal models verified that PPARα and PPARγ are able to exert anti-atherosclerotic effects *in vivo*. Clinical studies suggested that PPARα or PPARγ alone are capable of lowering plasma inflammatory cytokine secretion in T2DM patients with CAD (30–33). Our study revealed that RSG or BEZ alone was able to lower plasma CRP and MCP-1 levels in T2DM patients with CAD. However, the effect was clearer with combined RSG and BEZ, indicating that a combination of RSG and BEZ alleviates the inflammatory reaction more effectively. The mechanisms underlying the effect of RSG or BEZ on CRP and MCP-1 levels may include the modulation of NF-κB, AP-1 and signal transducer and activator of transcription (STAT) signal pathways. The present study demonstrated that a combination of RSG and BEZ exerts a more efficient anti-inflammatory effect than RSG or BEZ alone, indicating that a combination of PPARα/γ agonists is capable of inhibiting the expression of MCP-1, thus delaying the process of atherosclerosis.

In conclusion, our study demonstrated that a combination of RSG and BEZ is capable of reducing the level of blood glucose, alleviating IR and improving lipid modulation. A combination of RSG and BEZ is also able to lower plasma CRP and MCP-1 levels and decrease low-dose LPS-induced MCP-1 secretion by PBMCs in T2DM patients. The effects of combined RSG and BEZ are greater than those of RSG or BEZ used alone. The results of the present study suggest that the combination of PPARα/γ agonists may be more effective for the treatment of T2DM patients with CAD.

## Figures and Tables

**Table I.** Clinical and metabolic characteristics before treatment.

Variables	RSG group	BEZ group	Combination group	Control group	P-value
Male/female (n)	14/6	13/7	12/8	14/6	0.535
Age (years)	60.6±9.61	62.8±9.41	62.9±9.00	60.4±8.46	0.715
Smoking (n)	5	6	5	6	0.275
Hypertension (n)	5	6	5	6	0.230
BMI (kg/m^2^)	25.19±4.01	24.79±3.50	24.97±3.03	24.37±2.80	0.407
FPG (mmol/l)	7.27±1.69	7.77±4.08	8.57±4.42	7.45±2.86	0.925
Fins (mU/l)	11.48±4.65	13.07±4.89	12.28±4.61	12.07±4.94	0.868
HOMA-IR	3.69±1.61	4.07±2.05	4.77±3.29	3.74±1.56	0.423
HbA1c (%)	7.84±1.58	7.58±2.01	8.05±2.10	7.49±1.83	0.582
TC (mmol/l)	4.73±0.92	4.43±0.81	4.49±0.77	4.52±1.07	0.541
TG (mmol/l)	1.92±0.98	2.06±1.25	2.07±1.70	2.06±1.10	0.714
HDL (mmol/l)	1.04±0.18	1.04±0.21	0.94±0.28	1.05±0.24	0.267
LDL (mmol/l)	2.85±0.82	2.55±0.78	2.65±0.75	2.73±0.96	0.674
CRP (mg/l)	8.87 (5.47–15.91)	18.11 (7.71–34.44)	13.32 (4.22–21.06)	10.05 (4.73–18.64)	0.922
MCP-1 (pg/ml)	174.8 (83.2–223.3)	199.2 (70.3–297.3)	188.6 (84.1–253.2)	179.5 (65.9–241.6)	0.476

RSG, rosiglitazone; BEZ, bezafibrate; BMI, body mass index; FBG, fasting blood glucose; Fins, fasting insulin; HOMA-IR, insulin resistance by homeostasis model assessment; HbA1c, hemoglobin A1c; TC, total cholesterol; TG, triglyceride; HDL, high-density lipoprotein; LDL, low-density lipoprotein; CRP, C-reactive protein; MCP-1, monocyte chemoattractant protein-1.
